# Association of pain and quality of life among middle-aged and older adults of India

**DOI:** 10.1186/s12877-022-03480-y

**Published:** 2022-12-06

**Authors:** Amit Kumar Goyal, Sanjay K Mohanty

**Affiliations:** Department of Population and Development, International Institute for Population Sciences, Mumbai, India

**Keywords:** Pain, Epidemiology, Middle-aged adults, Older adults, Quality of life

## Abstract

**Background:**

India is passing through a phase of demographic and epidemiological transition where ageing and chronic morbidities are being more common. Though studies have examined the prevalence and risk factors of pain and other chronic morbidities, nationally representative research examining the association of pain and quality of life (QoL) is limited in India. This study examines the association between pain and QoL among middle-aged and older adults in India.

**Methods:**

This study uses the data from wave 1 of the Longitudinal Ageing Study in India (LASI) conducted in 2017-18. This study is restricted to 58,328 individuals from all states (except Sikkim), aged 45 years and above. The quality of life is measured in 6 domains (physical, psychological, social, environment, general health and life satisfaction) with 21 variables that range from 0 to 100. The principal component analysis was used to generate a composite score of QoL and the multiple linear regression was used to show the association between pain and quality of life.

**Results:**

It is estimated that approximately 37% of Indian middle-aged and older populations were often troubled with pain. Pain prevalence increase with age and is more common among older adults aged 75 + years (43.37%; 95% CI, 40.95–45.80), and female (41.38%; 95% CI, 39.36–43.39). The average QoL score among those with pain was 81.6 compared to 85.2 among those without pain. QoL was lower among elderly age 75 and above, females, rural residents and illiterates. Controlling for socio-demographic factors, pain reduces the QoL by 2.57 points (β= −2.57; 95% CI, −3.02 - −2.11).

**Conclusion:**

Pain reduces the quality of life among middle-aged adults and older adults in India. This evidence could potentially help the policymakers to consider pain as a significant determinant of quality of life in India.

**Supplementary information:**

The online version contains supplementary material available at 10.1186/s12877-022-03480-y.

## Background

Pain is a major public health challenge globally. One in every five adults worldwide suffers from pain, and one in every ten adults is diagnosed with chronic pain each year [1]. Despite its severity, it has been a neglected public health agenda. Though the pain has been widely discussed in medical literature demonstrating the biological and physiological domains of pain in developed countries, there is limited research highlighting the social and economic cost of pain in India. This void of research can be attributed to the recognition of pain as a mere symptom rather than a disease in itself and the lack of a standard definition of pain.

The International Association for the Study of Pain (IASP) defined pain as “an unpleasant sensory and emotional experience associated with or resembling that associated with, actual or potential tissue damage [2]. Pain can broadly be categorised into two types i.e. acute and chronic often distinguished based on duration. If pain persists for more than three months, it is termed as chronic pain and if it persists for less than 3 months it is termed as acute pain. Recently, the WHO recognised pain as a distinct disease in its 11th revision of the International Classification of Diseases (ICD-11) [3] and it led to a significant number of research publications on diverse dimensions of pain worldwide. In Germany, the prevalence of chronic pain was reported as 18.4% [4], while it was 21.5% in Hong Kong [5], 24.4% in Norway [6], 19% in Denmark [7] and 20.4% in the United States [8]. The distribution is relatively more uneven in developing countries ranging from 13–51% [9].

Pain is perceived as one of the most common health problems for older adults worldwide and is likely to result in lower quality of life [10–12]. Studies have shown that pain severely affects almost all segments of life i.e. sleep, ability to exercise, perform household chores, walk, attend social affairs and maintain independent lifestyles [13, 14]. and may lead to depression or anxiety [15–18]. Individuals suffering from pain are also vulnerable to substance abuse and other mental health issues [19, 20]. Besides being a serious health issue for individuals, pain imposes a significant social and economic burden [21] on households due to increased treatment costs and losses in quality of life. A Canadian study reported that half of the pain patients responded their condition had kept them from attending social or family events [22]. In Europe, almost half of the individuals with pain symptoms had less contact with their families [23]. The main cause of their social limitations was identified as difficulties in planning social activities due to the unpredictable nature of pain [24].

India with 254 million middle-aged and older adults is experiencing a shift in disease patterns. Non-communicable diseases (NCDs) are the leading cause of mortality and hospitalisation in the country. Older adults have very little social support system and the public health care system is not designed to treat NCDs. While an increasing number of studies are available on various NCDs among older adults, studies on pain in India are very limited. Despite the ubiquity of pain, whether acute, chronic or intermittent, public health research in India has not addressed this issue. The importance of viewing pain through a public health lens allows one to understand pain as a multifaceted, interdisciplinary problem for which many of the causes are the social determinants of health. To our knowledge, a limited number of micro-level studies have been conducted to estimate the prevalence of chronic pain [25–27] among Indian adults and associated risk factors of pain among specific population groups [28–30]. A recent study estimated that around 36.6% of the Indian population aged 45 + have often troubled with pain [31]. We didn’t find any population representative studies that examine the association between pain and quality of life among middle-aged and older adults in India. This study examines the association between pain and quality of life among the middle-aged and older population of India using a nationally representative survey.

## Methods

### Study design and participants

**Fig. 1:**
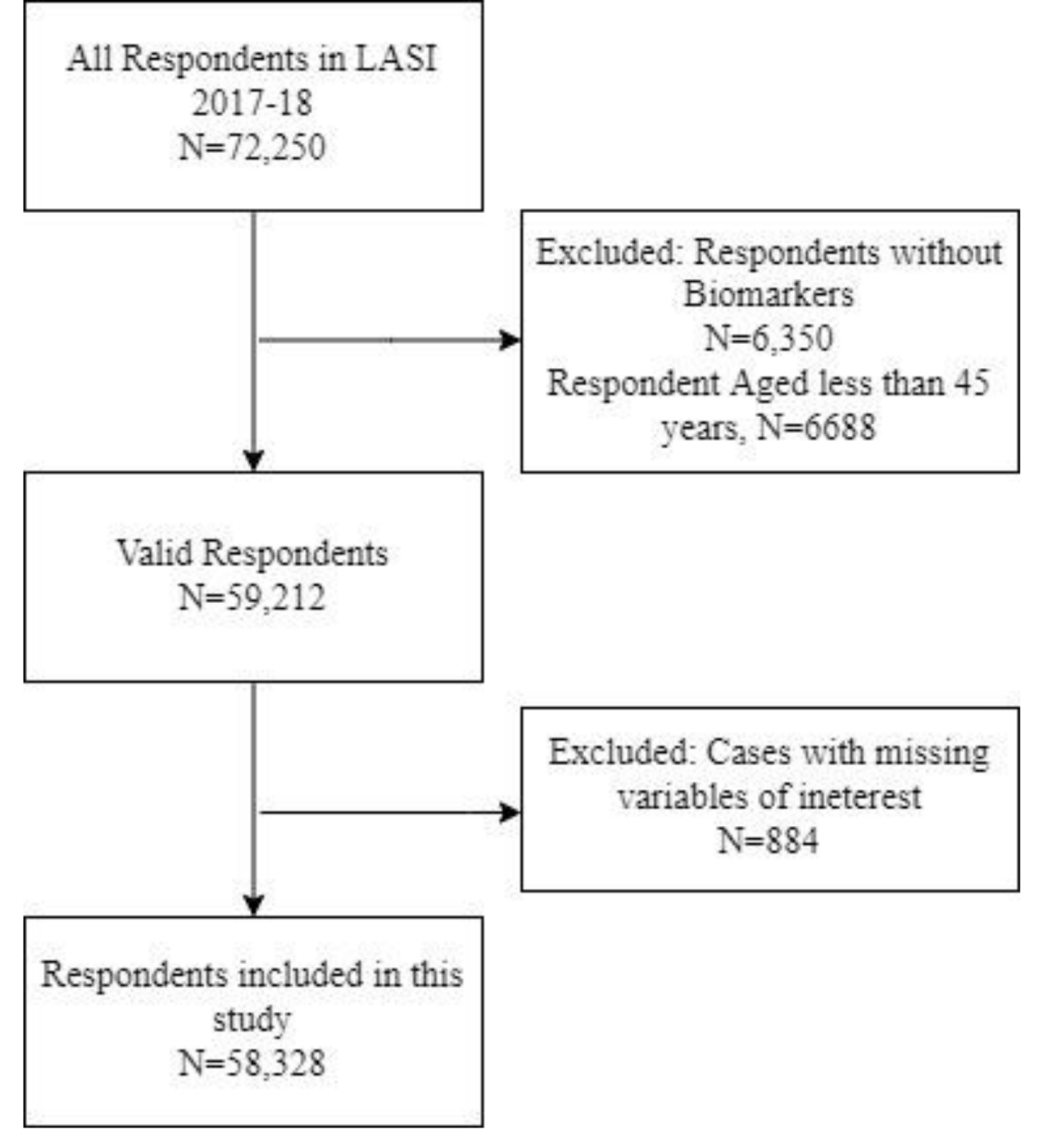
Flowchart for the sample design

This study is based on a cross-sectional study design. The data has been extracted from wave 1 of the Longitudinal Ageing Study in India (LASI) conducted in 2017-18. LASI is a nationally representative prospective cohort of all Indian adults and older men and women age 45 and above and their spouses who reside in the same household, irrespective of age. LASI Wave 1 collected data from all 36 states and union territories (data of Sikkim was not available at the time of submission of this paper). LASI used stratified, multistage cluster sampling to select (non-institutional) households, within which all individuals aged 45 years and older, and their spouses, were interviewed face-to-face [32]. This study is restricted to only 58,328 individuals from all states (except Sikkim), who were aged above 45 years and above and responded to all the variables of interest for this study (Fig. 1).

### Ethical approval

for the study was obtained from the Health Ministry’s Screening Committee (Government of India) and the Institutional Review Boards (IRB) at the International Institute for Population Sciences (IIPS) and its collaborating institutions including the Indian Council of Medical Research (ICMR), Delhi; IRB, Harvard T.H. Chan School of Public Health (HSPH), Boston; IRB, University of Southern California (USC), Los Angeles; IRB, ICMR National AIDS Research Institute (NARI), Pune; and IRB, Regional Geriatric Centres (RGCs), MoHFW. Written informed consents were obtained from all study participants [32].

### Measures:

#### Quality of life

The World Health Organisation (WHO) defines QoL as “an individual’s perception of their position in life in the context of the culture and value systems in which they live and about their goals, expectations, standards and concerns.” [33]. In general terms, it refers to the evaluation of the general well-being of an individual. A composite index was formulated by using Principal Component Analysis (PCA) to measure the QoL. It consists of 21 items in six domains i.e. physical health, psychological health, social relationship, environmental satisfaction, life satisfaction and general health [34, 35]. The QoL score and each domain score were transformed into a linear scale between 0 and 100. A higher score indicated a better quality of life and vice-versa. Appendix 2 presents the variables used in constructing the quality of life, their mean and factor score.

Physical health was assessed by considering the Activities of Daily Living (ADL) (dressing, bathing, walking, eating, getting out of bed, using the toilet), physical energy and sleep comfort. Psychological wellbeing was examined through self-reported inner peace, positive and negative feelings, satisfaction, spirituality and concentration ability. The environmental aspect of QoL was assessed by their financial status, feeling about their safety and satisfaction with living arrangements. The social domain was examined by living arrangements and the number of friends. Life satisfaction and general health were evaluated by individual questions. All questions were recoded into dichotomous variables for further statistical analysis (Appendix 1).

#### Pain

The overall sample was grouped into two categories i.e., respondents with pain and without pain. Those participants responded affirmatively to the question *Are you often troubled with pain?* were categorised as respondents ‘with pain” or else ‘without pain’(Appendix 1).

#### Covariates

Several factors that have been identified as potential confounders in the relationship between QoL and pain were included in the multiple regression model. Demographic characteristics included age groups (“45–59”, “60–74”, and “75 and above”), sex (“male” and “female”), residence (“rural” and “urban”), an education level (“no schooling”, “less than 5 years”, “5–9 years” and “10 and more years of schooling”), currently married (“yes”, and “no”). Economic status was indicated by household wealth quintiles (“poorest”, “poorer”, “middle”, “richer”, “richest”), work status (“currently working”, “ever worked but not currently working” and “never worked”). Health aspect was assessed by several comorbidities (“0” and “≥1”), BMI level (“underweight”, “normal”, “overweight” and “obese”), and three dichotomous covariates like dependence on any aids, history of smoking and alcohol consumptions.

#### Data analysis

Descriptive statistics were reported by proportions or mean and confidence intervals. Independent chi-square tests were used to compare the categorical variables between those with and without pain. The estimates of pain prevalence and quality of life were adjusted for age and sex fixed effects (supplementary text 1). The Multiple Linear Regression (MLR) model was used with the QoL score as the dependent variable and pain and socio-demographic covariates as independent variables. STATA 16.1 and ArcMap 10.8 have been used to perform all the statistical analysis and map visualizations respectively [36]. We used a significance threshold of p < 0.05.

## Results

### Baseline characteristics

LASI survey included 72,250 individuals and data on Sikkim was not publicly available. This study is based on 58,328 respondents who had responded to all the variables of interest for this study. Table 1 shows the distribution of the sample population by their socio-demographic attributes. Approximately 37% of the sample population have reported pain. The majority of them were middle-aged adults (33.4%), female (60.9%), resided in rural areas (68.8%), and had no education (51.4%). About 72.5% were currently married, 42.6% were engaged in active working, 53.9% were multi-morbid, 51.2% had normal BMI and 61% and 82.3% had never smoked and drunk alcohol respectively at the time of the survey. The chi-square test shows the distributions are significantly different between the two pain groups (Table 1).

**Table 1:** Socio-demographic characteristics of middle-aged and older adults with and without pain (N = 58,328), India, 2017-18

Socio-Demographic Characteristics	Pain		*p-value*
	**Yes (N = 21,650)**	**No (N = 36,678)**	
	**M ± SD or N (%)**	**M ± SD or N (%)**	
**Age**			< 0.001
45–59	10,457 (48.3)	20,208 (55.1)	
60–74	8745 (40.4)	13,317 (36.3)	
75 and above	2448 (11.3)	3153 (8.6)	
	60.54 ± 10.7	58.93 ± 10.3	
**Sex**			< 0.001
Male	8470 (39.1)	18,484 (50.4)	
Female	13,180 (60.9)	18,194 (49.6)	
**Residence**			< 0.001
Rural	14,884 (68.8)	23,307 (63.5)	
Urban	6766 (31.3)	13,371 (36.5)	
**Years of Education**			< 0.001
No schooling	11,132 (51.4)	16,271 (44.4)	
< 5 years	2726 (12.6)	4074 (11.1)	
5–9 years	4791 (22.1)	8572 (23.4)	
≥ 10 years	3001 (13.9)	7761 (21.2)	
**Currently Married**			< 0.001
Yes	15,697 (72.5)	28,006 (76.4)	
No	5953 (27.5)	8672 (23.6)	
**MPCE quintile**			0.481
Poorest	4275 (19.8)	7241 (19.7)	
Poorer	4419 (20.4)	7396 (20.2)	
Middle	4371 (20.2)	7407 (20.2)	
Richer	4406 (20.4)	7342 (20.0)	
Richest	4179 (19.3)	7292 (19.9)	
**Work Status**			< 0.001
Currently Working	9220 (42.6)	17,983 (49.0)	
Ever worked but not Currently Working	6109 (28.2)	9035 (24.6)	
Never Worked	6321 (29.2)	9660 (26.3)	
**Multi-morbidity**			< 0.001
0	9986 (46.1)	21,301 (58.1)	
≥ 1	11,664 (53.9)	15,377 (41.9)	
**BMI**			< 0.001
Underweight (≤ 18.5)	4110 (19.0)	6713 (18.3)	
Normal (18.5–25.0)	11,082 (51.2)	19,397 (52.9)	
Overweight (25.0–30.0)	4756 (22.0)	7948 (21.7)	
Obese (> 30)	1702 (7.9)	2620 (7.1)	
	22.83 ± 4.9	22.80 ± 4.7	
**Smoking History**			< 0.001
No	13,213 (61.0)	23,558 (64.2)	
Yes	8437 (39.0)	13,120 (35.8)	
**Alcohol History**			0.059
No	17,823 (82.3)	29,966 (81.7)	
Yes	3827 (17.7)	6712 (18.3)	
**Supportive Aid for Daily Life**			< 0.001
Independent	12,617 (58.3)	23,004 (62.7)	
Uses Aid	9033 (41.7)	13,674 (37.3)	

Note: MPCE refers to monthly per capita consumption expenditure and BMI refers to body mass index

### Pain prevalence

Table 2 shows the age-sex adjusted prevalence of pain by socio-demographic characteristics. 36.5% of Indian middle-aged and older adults have often troubled by pain. It is more common among older adults above 75 years (43.37; 95% CI, 40.95–45.80), females (41.38; 95% CI, 39.36–43.39), rural residents (38.74; 95% CI, 37.66–39.83), had less than 5 years of education (42.16; 95% CI, 40.11–44.26), currently married (37.53; 95% CI, 36.26–38.80), retired individuals (40.25; 95% CI, 38.74–41.77), had multimorbid conditions (41.89; 95% CI, 40.02–43.76).

**Table 2:** Age and sex adjusted pain prevalence among middle-aged and older adults of India by socio-demographic characteristics, India, 2017-18

Socio-demographic Characteristics	Adjusted Prevalence
	**% (95% CI)**
**Overall**	36.54 (35.16–37.91)
**Age**	
45–59	33.29 (31.74–34.83)
60–74	38.86 (37.16–40.56)
75 and above	43.37 (40.95–45.80)
**Sex**	
Male	30.78 (29.66–31.90)
Female	41.38 (39.36–43.39)
**Residence**	
Rural	38.74 (37.66–39.83)
Urban	31.36 (28.08–34.64)
**Years of Education**	
No schooling	37.69 (36.29–39.10)
< 5 years	42.19 (40.11–44.26)
5–9 years	36.70 (34.55–38.86)
≥ 10 years	28.91 (24.91–32.90)
**Currently Married**	
Yes	37.53 (36.26–38.80)
No	33.95 (31.65–36.25)
**MPCE quintile**	
Poorest	37.51 (35.60–39.41)
Poorer	37.09 (35.37–38.80)
Middle	36.76 (34.84–38.67)
Richer	36.31 (33.84–38.78)
Richest	34.69 (31.74–37.63)
**Work Status**	
Currently Working	36.66 (35.30–38.01)
Ever worked but not Currently Working	40.25 (38.74–41.77)
Never Worked	32.77 (29.68–35.87)
**Multi-morbidity**	
0	31.90 (30.52–33.29)
≥ 1	41.89 (40.02–43.76)
**BMI**	
Underweight (≤ 18.5)	36.61 (34.94–38.28)
Normal (18.5–25.0)	36.83 (35.63–38.02)
Overweight (25.0–30.0)	36.06 (32.92–39.19)
Obese (> 30)	35.61 (31.86–39.36)
**Smoking History**	
No	33.00 (31.31–34.69)
Yes	42.79 (41.43–44.14)
**Alcohol History**	
No	35.83 (34.38–37.28)
Yes	40.81 (38.81–42.82)
**Supportive Aid for Daily Life**	
Independent	34.39 (33.03–35.75)
Uses Aid	40.05 (37.76–42.34)

Note: See Supplementary File for method of age and sex adjustment. Estimates by sex were adjusted by age composition only. Estimates by age group were adjusted for sex composition only. MPCE refers to monthly per capita consumption expenditure and BMI refers to body mass index

### Quality of life among middle-aged and older adults of India

Table 3 shows the mean scores of QoL along with its domains among older adults with and without pain. The mean QoL score was 85.2 (SD = 11.4) among adults without pain whereas it was 81.6 (SD = 13.6) among adults with pain. All other domains except the social domain follow a similar pattern where QoL drops significantly with the occurrence of pain.

**Table 3:** Estimated scores of Quality of Life and its domains by pain among middle-aged and older adults

Domains	Pain				p-value	Total	
	**Yes (N = 21,650)**		**No (N = 36,678)**				
	**Mean (SD)**	**95% CI**	**Mean (SD)**	**95% CI**		**Mean (SD)**	**95% CI**
General Health	74.00 (43.86)	73.42–74.58	88.74 (31.61)	88.41–89.06	< 0.001	83.27 (37.33)	82.96–83.57
Life Satisfaction	87.70 (32.84)	87.27–88.14	91.96 (27.19)	91.68–92.24	< 0.001	90.38 (29.49)	90.14–90.62
Physical QoL	87.43 (16.71)	87.21–87.66	93.26 (11.75)	93.14–93.38	< 0.001	91.10 (14.08)	90.98–91.21
Psychological QoL	71.71 (22.70)	71.41–72.01	72.24 (22.81)	72.01–72.47	< 0.001	72.04 (22.77)	71.86–72.23
Social QoL	80.20 (19.15)	79.94–80.45	79.71 (18.45)	79.52–79.90	0.002	79.89 (18.71)	79.74–80.04
Environmental QoL	93.60 (15.66)	93.39–93.81	95.27 (13.18)	95.13–95.40	< 0.001	94.65 (14.17)	94.54–94.77
**Overall Quality of Life**	**81.60 (13.60)**	**81.42–81.78**	**85.16 (11.36)**	**85.05–85.28**	**< 0.001**	**83.84 (12.36)**	**83.74–83.94**

Table 4 shows the adjusted scores of QoL among Indian adults with and without pain by socio-demographic characteristics. The scores were adjusted for age and sex fixed effects. It is found that the occurrence of pain reduces the QoL scores for all sociodemographic characteristics. The QoL also declines with increasing age irrespective of pain. Among the population with pain, the QoL was found relatively better among males 82.43; (95% CI, 81.87– 82.99), urban residents (82.46; 95% CI, 81.85–83.07), who had 10 years of education (83.51; 95% CI, 82.76–84.26), were married (82.32; 95% CI, 81.83–82.80) and working 82.05; 95% CI, 81.47–82.63), and had no morbid condition (82.20; 95% CI, 81.68–82.72) in their respective categories.

**Table 4:** Age-sex adjusted scores of Quality of Life among middle-aged and older adults with and without pain by socio-demographic characteristics in India, 2017-18

Socio-Demographic Characteristics	Pain	
	**Yes (N = 21,650)**	**No (N = 36,678)**
	**Mean QoL Score (95% CI)**	**Mean QoL Score (95% CI)**
**Age**		
45–59	83.28 (82.86–83.71)	86.08 (85.75–86.40)
60–74	80.13 (79.59–80.67)	83.42 (82.85–84.00)
75 and above	73.95 (72.25–75.65)	80.69 (79.85–81.52)
**Sex**		
Male	82.43 (81.87–82.99)	85.27 (84.91–85.63)
Female	79.79 (79.31–80.28)	83.86 (83.34–84.38)
**Residence**		
Rural	80.25 (79.70–80.79)	83.79 (83.44–84.14)
Urban	82.46 (81.85–83.07)	86.17 (85.49–86.85)
**Years of Education**		
No schooling	79.85 (79.25–80.45)	83.51 (83.08–83.94)
< 5 years	80.78 (80.01–81.56)	84.04 (83.37–84.72)
5–9 years	81.80 (81.06–82.53)	85.15 (84.56–85.73)
≥ 10 years	83.51 (82.76–84.26)	86.71 (86.11–87.31)
**Currently Married**		
Yes	82.32 (81.83–82.80)	85.50 (85.12–85.88)
No	76.87 (76.17–77.58)	81.79 (81.12–82.46)
**MPCE quintile**		
Poorest	80.88 (80.11–81.65)	84.37 (83.84–84.90)
Poorer	81.37 (80.59–82.16)	84.29 (83.68–84.91)
Middle	80.73 (79.91–81.56)	84.58 (84.07–85.10)
Richer	80.61 (79.89–81.32)	84.76 (84.19–85.33)
Richest	80.31 (79.50–81.13)	84.83 (83.81–85.85)
**Work Status**		
Currently Working	82.05 (81.47–82.63)	84.67 (84.28–85.07)
Ever worked but not Currently Working	78.84 (78.18–79.51)	82.86 (82.28–83.44)
Never Worked	81.06 (80.26–81.87)	85.94 (85.25–86.63)
**Multi-morbidity**		
0	82.20 (81.68–82.72)	85.20 (84.86–85.55)
≥ 1	79.62 (79.05–80.19)	83.64 (83.03–84.26)
**BMI**		
Underweight (≤ 18.5)	79.02 (78.14–79.90)	82.58 (82.04–83.11)
Normal (18.5–25.0)	80.99 (80.47–81.52)	84.55 (84.22–84.88)
Overweight (25.0–30.0)	82.08 (81.52–82.64)	85.90 (85.14–86.66)
Obese (> 30)	81.49 (80.32–82.66)	86.72 (85.42–88.02)
**Smoking History**		
No	81.64 (81.12–82.16)	85.11 (84.59–85.63)
Yes	79.58 (78.94–80.22)	83.59 (83.20–83.97)
**Alcohol History**		
No	80.86 (80.39–81.33)	84.63 (84.22–85.04)
Yes	80.54 (79.75–81.32)	84.15 (83.62–84.69)
**Supportive Aid for Daily Life**		
Independent	80.82 (80.33–81.32)	84.30 (83.93–84.66)
Uses Aid	80.80 (80.17–81.42)	85.03 (84.29–85.76)

Note: See Supplementary File for method of age and sex adjustment. Estimates by sex were adjusted by age composition only. Estimates by age group were adjusted for sex composition only. MPCE is an abbreviation for monthly per capita consumption expenditure and BMI refers to body mass index

In Table 5, the results of the regression model show the relationship between pain and other socio-demographic characteristics on QoL suggests that pain reduces the QoL by 2.57 points (β= −2.57; 95% CI, −3.02 – −2.11). Apart from pain, socio-demographic predictors also affect the QoL among adults aged 45 and above. The QoL declines with an increase in age, it reduces by around three points at the age of 75 and above (β= −2.80; 95% CI, −3.65 – −1.95). Older female has lower QoL than their male counterparts (β= −0.57; 95% CI, −1.05 – −0.10). Those adults reside in urban area (β = 1.95; 95% CI, 1.37–2.53), had 10 or more years of education (β = 2.43; 95% CI, 1.85–3.00), obese (β = 2.09; 95% CI, 1.11–3.06) had relatively better QoL. On the other hand, those who were retired (β= −2.24; 95% CI, −2.74 – −1.75), had multi-morbid conditions (β= −2.23; 95% CI, −2.62 – −1.84) and smoking history (β= −0.91; 95% CI, −1.41 – −0.40) experience relatively poor QoL.

**Table 5:** Multiple linear regression of the potential factors associated with the overall QoL of middle-aged and older adults. (N = 58,328)

Factors	β	95% CI	p-value
**Pain**			
No ®	-		
Yes	−2.57	−3.02 – −2.11	< 0.001
**Age**			
45–59®	-		
60–74	−0.76	−1.19 – −0.34	< 0.001
75 and above	−2.8	−3.65 – −1.95	< 0.001
**Sex**			
Male®	-		
Female	−0.57	−1.05 – −0.10	0.018
**Residence**			
Rural	-		
Urban	1.95	1.37–2.53	< 0.001
**Education Level**			
No schooling®	-		
< 5 years	0.92	0.37–1.46	0.001
5–9 years	1.41	0.86–1.95	< 0.001
≥ 10 years	2.43	1.85–3.00	< 0.001
**Currently Married**			
Yes®	-		
No	−4.21	−4.65 – −3.78	< 0.001
**MPCE quintile**			
Poorest®	-		
Poorer	0.19	−0.45–0.82	0.564
Middle	−0.27	−0.82–0.29	0.342
Richer	−0.38	−0.97–0.21	0.206
Richest	−0.79	−1.46 – −0.12	0.021
**Work Status**			
Currently Working®	-		
Ever worked but not Currently Working	−2.24	−2.74 – −1.75	< 0.001
Never Worked	−0.32	−0.88–0.25	0.269
**Multi-morbidity**			
0®	-		
≥ 1	−2.23	−2.62 – −1.84	< 0.001
**BMI**			
Underweight®	-		
Normal	1.51	1.05–1.98	< 0.001
Overweight	2.2	1.60–2.80	< 0.001
Obese	2.09	1.11–3.06	< 0.001
**Smoking History**			
No®	-		
Yes	−0.91	−1.41 – −0.40	< 0.001
**Alcohol History**			
No®	-		
Yes	−0.02	−0.51–0.47	0.942
**Supportive Aid for Daily Life**			
Independent®	-		
Uses Aid	−0.22	−0.79–0.36	0.456

Note: adjusted for all covariates listed in Table 1 and state-level fixed effect. MPCE refers to monthly per capita consumption expenditure and BMI refers to body mass index

**Fig. 2:**
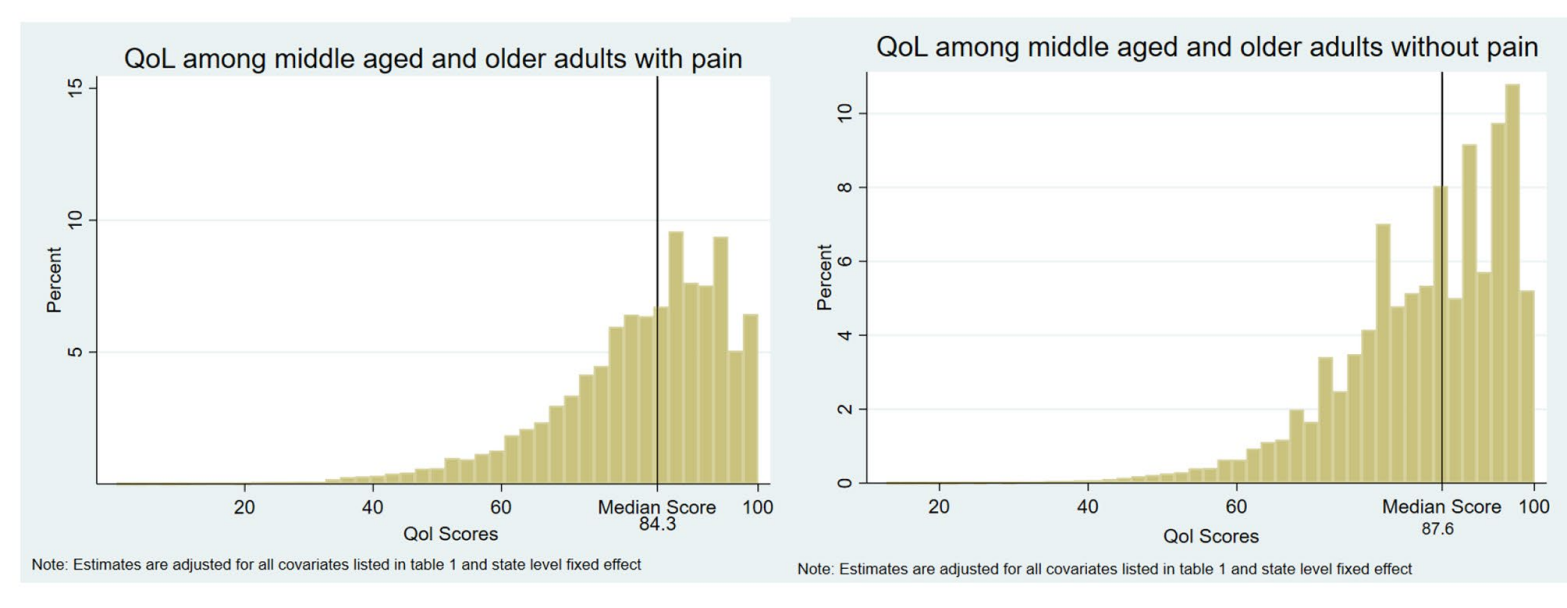
(a) and (b): Distribution of QoL among middle-aged and older adults with and without pain

Figure 2a and b show the distribution by QoL among older adults with and without pain respectively. In the absence of pain, the median QoL score among middle-aged and older adults was found 87.6 whereas it reduces to 83.4 in the case of the population with pain.

**Fig. 3:**
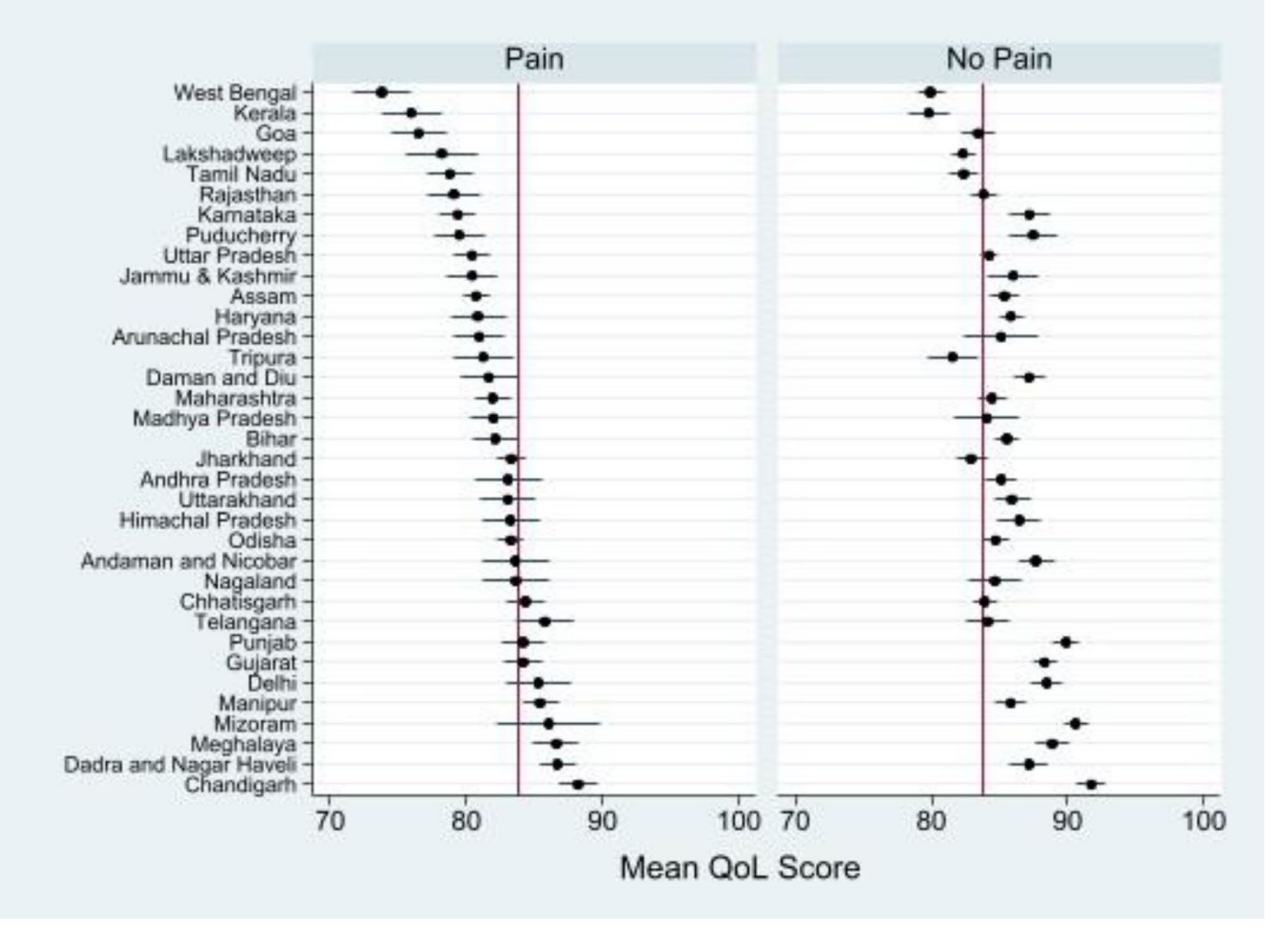
State-level adjusted estimates of QoL among middle-aged and older adults of India

Figure 3 depicts the state-level estimates of QoL by pain among middle-aged and older adults. Among the adults with pain, West Bengal, Kerala and Goa had the lowest QoL whereas the union territories i.e. Chandigarh and Dadar & Nagar Haveli had the highest level QoL followed by states like Meghalaya and Mizoram.

**Fig. 4:**
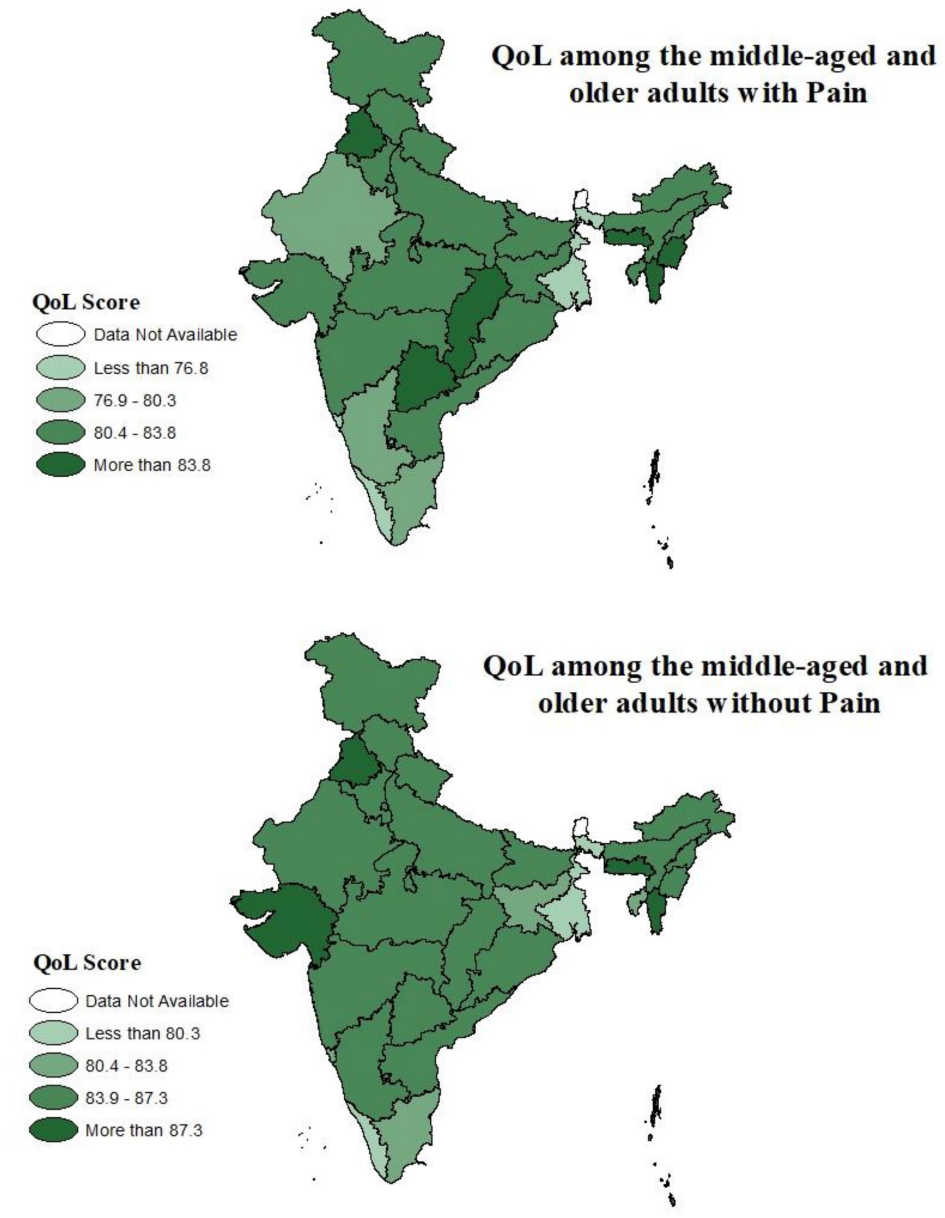
Spatial distribution of adjusted QoL among middle-aged and older adults with and without pain

Figure 4 depicts the spatial distribution of older adults with and without pain. Concerning the average QoL score., it is found that in the absence of pain 28 of 36 states/UTs (for which data were analysed) had a better QoL than average score whereas those with pain had a lower QoL score. There are exceptions in a few states of India like Punjab, Chhattisgarh, Telangana, Meghalaya, Mizoram and Manipur have better QoL.

## Discussion

To the best of our knowledge, this is the first nationally representative study that examined the association between pain and quality of life among middle-aged and older adults in India. This study also presents potential factors for understanding and improving quality of life by exploring the relationships between socio-demographic characteristics, pain and QoL among middle-aged and older adults of India. We provide the following possible explanations in support of our results.

Our estimates of the pain prevalence at 36.5% are consistent with the global and regional pain surveys ranging from 30 to 40% [13, 37–40]. Our findings are also consistent with the earlier estimates of pain prevalence among Indian adults aged 45 and above [31]. The pain prevalence was found higher among the older adults [25, 26], females [25], rural residents, illiterates [41] and the retired population. It also validates the findings of Cabral et al. which shows that pain is more common among multi-morbid older adults [42] and those with poor lifestyle habits like smoking and alcohol consumption lead to sustainable pain [43].

Our finding that pain is associated with poorer QoL among adults above 45 years of age is also consistent with previous findings [15, 44]. Those with pain had a consistency of lower QoL than those without pain in each domain (except social). The differences in QoL were the largest in physical health and lowest in the psychological aspects. Because it becomes very difficult for the patients to perform activities of daily living, their sleep quality deteriorates, they usually feel depressed [45, 46]. It is also evident that older adults with pain usually rate relatively lower life satisfaction due to various limiting factors including severe health and environmental problems [47]. Varying factors like kinesiophobia, fear avoidance belief, or pain belief; occupation-related factors; pain and disability; disease; activity; and lack of pain treatment also reduces the QoL among adults with pain [48]. As pain treatment is very limited in India and over one-fourth of middle-aged and older adults with pain do not use any medication [31].

Our findings are consistent with the earlier findings that older women with pain experience relatively poorer QoL than their male counterparts. The poorer QoL in women may be due to the prevalence of a higher rate of nonfatal disabled disorders and the difference in the perceived health between the sexes. Moreover, the reporting of pain is relatively higher among women than men [35, 49]. Our regression results also confirms that the QoL among rural residents is relatively poor, as earlier studies have shown that older people in rural areas have poorer physical and mental health than those in urban areas [50]. Furthermore, when compared to their urban counterparts, older rural residents experience more social isolation and report lower social functioning [51, 52]. We have also found significantly better QoL among currently married older adults as married people usually have improved mental health compared with those who are single, divorced, or bereaved due to the social relationship with the spouse [53, 54]. On the line of previous findings, we also found that the QoL is relatively better among educated [55, 56]. Our study also supports the findings of Selvamani et al. (2018) which showed significantly lower QoL among underweight and relatively better quality of life among overweight older adults of India [56]. This might be due to the fact that overweight or obese people usually have a poor physical function but good psychological health conditions [57]. Moreover, the overweight and obesity in Indian context is generally higher among richer and socially advantages section of the population. These group of population are likely to have better psychological, social and environmental QoL compared to those who are underweight. The QoL among working older adults are also found to be significantly better in all domains of quality of life than retire or unemployed older adults [35].

The spatial distribution of the QoL score identifies the zones where the average score is relatively better than the national average. For instance, Punjab & Chandigarh in the northwest, Chhattisgarh and Telangana in the central-east part and Manipur, Meghalaya and Mizoram in the north-eastern part of India. It is also worth noting that more than 25 state/UTs had lower than average QoL considering the adults aged 45 + with pain.

There are a few strengths and limitations of this study as well. The strength of this study lies in the utilization of a large nationally representative survey dataset that provides reliable estimates for India and its states. Limitations of this study include, the cross-sectional data from a longitudinal cohort study which doesn’t allow us to interpret the causation; and, the use of the open-ended question to measure pain in the absence of scale, though previous studies have used the same measure to estimate its prevalence [31].

## Conclusion

This study provides insight into the association between pain and the quality of life among adults aged 45 and above, calling for greater attention to the effectiveness of pain management. This study shows that over one-third of adults aged 45 and above in India had experienced pain and it is negatively associated with quality of life. Assuming the ageing trend over the years, the prevalence may increase significantly during the next three decades, negatively impacting the living standard of India’s senior citizens. This study also adds to the evidence that along with various sociodemographic factors, pain is also a significant contributor to the quality of life of older adults. To achieve the goal of successful ageing, subjective well-being, life satisfaction and happiness among middle-aged and older adults of India, it should draft policies after considering pain as a significant contributor to it.

## Electronic supplementary material

Below is the link to the electronic supplementary material.

Supplementary Material 1.
Appendix 1: Variables and scores for Index of Quality of life and Pain measurement.
Appendix 2: Principal Component Analysis results for Index of Quality of life.
Supplementary Text 1: Age sex adjustment method.

## Data Availability

The datasets generated during and/or analyses during the current study are available from the corresponding author upon reasonable request.
