# Supplementary material for: Association of pain and quality of life among middle-aged and older adults of India
Source: BMC Geriatr. 2022 Dec 6;22:939. doi: 10.1186/s12877-022-03480-y (PMC9724285; doi:10.1186/s12877-022-03480-y)
Supplement: Supplementary file 1 — Supplementary Material 1. Appendix 1: Variables and scores for Index of Quality of life and Pain measurement. Appendix 2: Principal Component Analysis results for Index of Quality of life. Supplementary Text 1: Age sex adjustment method. [file 12877_2022_3480_MOESM1_ESM.docx]

| Appendix 1: Variables and scores for Index of Quality of life and Pain measurement. | | | | | | | | | |
| --- | --- | --- | --- | --- | --- | --- | --- | --- | --- |
| **Variables** | | **Question** | | | | | **Score** | | |
| **Physical Domain** | | **8 Items** | | | | |  | | |
| **ADLs** | |  | | | | |  | | |
|  | | Any difficulty with these because of a physical, mental, emotional, or memory problem…. | | | | |  | | |
| Dressing | | Dressing, including putting on chappals, shoes, etc | | | | | Yes=0, No=1 | | |
| Bathing | | Bathing | | | | | Yes=0, No=1 | | |
| Walking | | Walking across a room | | | | | Yes=0, No=1 | | |
| Eating | | Eating, difficulties | | | | | Yes=0, No=1 | | |
| Bedding | | Getting in or out of bed | | | | | Yes=0, No=1 | | |
| Toilet | | Using the toilet, including getting up and down | | | | | Yes=0, No=1 | | |
| Physical Energy | | Did you feel tired or low in energy during the past week? | | | | | Yes=0, No=1 | | |
| Sleep | | Did you experience trouble falling asleep during the past 1 month? | | | | | Yes=0, No=1 | | |
| **Psychological Domain** | | **6 Items** | | | | |  | | |
| Feeling of Inner Peace | | Do you think that you have a feeling of deep inner peace? | | | | | Yes=1, No=0 | | |
| Feel Happy | | Did you feel happy during the past week? | | | | | Yes=1, No=0 | | |
| Feel Depressed | | Did you feel depressed during the past week? | | | | | Yes=0, No=1 | | |
| Feel Overall Satisfied | | Did you feel you were overall satisfied during the past week? | | | | | Yes=1, No=0 | | |
| Feel Spiritual | | Do you think that you are spiritually touched by the beauty of creation? | | | | | Yes=1, No=0 | | |
| Feel Focused/Concentrated | | Did you have trouble concentrating during the past week? | | | | | Yes=0, No=1 | | |
| **Environment Domain** | | **3 Items** | | | | |  | | |
| Financial Status | | Have you received financial help/support from your family or friends during the past 12 months? | | | | | Yes=0, No=1 | | |
| Feeling safe | | Do you feel safe from crime and violence when you are alone at home? | | | | | Yes=1, No=0 | | |
| Satisfaction with living arrangement | | Are you satisfied with your current living arrangements? | | | | | Yes=1, No=0 | | |
| **Social Domain** | | **2 Items** | | | | |  | | |
| Have Friends | | Do you have a close relationship with your friends | | | | | Yes=1, No=0 | | |
| Living arrangement | | Do you live alone or with others? | | | | | Alone=0, With Others=1 | | |
| **Additional** | | **2 Items** | | | | |  | | |
| **Self-Reported Health/ General Health** | | How is your health in general? | | | | | Good=1, Poor=0 | | |
| **Life Satisfaction** | | Are you satisfied with your life? | | | | | Yes=1, No=0 | | |
| **Pain** | | Are you often troubled with pain? | | | | | Yes=1, No=0 | | |
|  |  | |  |  |  |  | |  |  |

| Appendix 2: Principal Component Analysis results for Index of Quality of life | | | | | | | |
| --- | --- | --- | --- | --- | --- | --- | --- |
| **Variables** | **M ± SD** | **PC1** | **PC2** | **PC3** | **PC4** | **PC5** | **PC6** |
| **Physical Domain** |  |  |  |  |  |  |  |
| **ADLs** |  |  |  |  |  |  |  |
| Dressing | 96.6 ± 18.2 | 0.3661 | -0.1085 | -0.1244 | -0.0111 | -0.0276 | 0.0370 |
| Walking | 96.8 ± 17.7 | 0.3923 | -0.1030 | -0.1395 | -0.0062 | -0.0361 | 0.0189 |
| Bathing | 96.8 ± 17.7 | 0.4065 | -0.1117 | -0.1415 | 0.0001 | -0.0236 | 0.0243 |
| Eating | 96.3 ± 18.9 | 0.3452 | -0.0877 | -0.1085 | 0.0173 | -0.0107 | 0.0070 |
| Bedding | 94.0 ± 23.8 | 0.3886 | -0.0819 | -0.0837 | -0.0329 | 0.0510 | -0.0042 |
| Toilet | 90.7 ± 29.1 | 0.3605 | -0.0790 | -0.0593 | -0.0173 | 0.0772 | -0.0128 |
| Physical Energy | 94.7 ± 22.5 | 0.1409 | 0.1227 | 0.3259 | -0.2641 | -0.3402 | 0.3368 |
| Sleep | 60.5 ± 48.9 | 0.1321 | 0.0122 | 0.2586 | 0.0139 | 0.1993 | -0.2223 |
| **Psychological Domain** |  |  |  |  |  |  |  |
| Feeling of Inner Peace | 80.4 ± 39.7 | 0.0641 | 0.5027 | -0.1552 | -0.3594 | 0.2784 | 0.0030 |
| Feel Happy | 60.9 ± 48.8 | 0.1225 | 0.0254 | 0.4197 | -0.1273 | 0.0396 | -0.1464 |
| Feel Depressed | 81.4 ± 38.9 | 0.0303 | 0.3417 | -0.2497 | 0.2913 | -0.2945 | 0.1492 |
| Feel Overall Satisfied | 61.7 ± 48.6 | -0.0061 | 0.2948 | -0.3524 | 0.3009 | -0.2562 | 0.1960 |
| Feel Spiritual | 71.6 ± 45.1 | 0.0592 | 0.4778 | -0.1792 | -0.3683 | 0.3248 | -0.0293 |
| Feel Focused/Concentrated | 62.6 ± 48.4 | 0.0994 | 0.1364 | 0.3254 | -0.2831 | -0.3920 | 0.3745 |
| **Environment Domain** |  |  |  |  |  |  |  |
| Financial Status | 86.7 ± 33.9 | 0.0395 | -0.0377 | 0.1287 | 0.2024 | 0.4139 | 0.5053 |
| Feeling safe | 95.6 ± 20.6 | 0.0746 | 0.1242 | 0.1840 | 0.0890 | -0.0303 | -0.1104 |
| Satisfaction with living arrangement | 95.8 ± 20.1 | 0.0916 | 0.2649 | 0.2559 | 0.3367 | 0.0068 | -0.1084 |
| **Social Domain** |  |  |  |  |  |  |  |
| Have Friends | 35.0 ± 47.7 | 0.0681 | 0.1639 | -0.0285 | -0.0347 | -0.3156 | -0.4814 |
| Living arrangement | 96.5 ± 18.5 | 0.0390 | 0.0877 | 0.1020 | 0.2632 | 0.2591 | 0.2509 |
| **Self-Reported Health/General Health** | 83.3 ± 37.3 | 0.1938 | 0.1424 | 0.1719 | 0.1991 | 0.0299 | -0.1533 |
| **Life Satisfaction** | 90.4 ± 29.5 | 0.1290 | 0.2889 | 0.2611 | 0.3453 | 0.0526 | -0.1179 |
| % Variance Explained |  | 17.9 | 9.4 | 8.9 | 5.9 | 5.2 | 4.8 |

**Supplementary Text 1: Age sex adjustment Method**

The following text was adapted from that appearing as Supplementary Text S4 in Mohanty et al. (2021) [1].

We adjusted estimates of pain prevalence and quality of life (QoL) for age and sex by using the age-sex composition of the nationally representative full sample as the reference. For example, to obtain age-sex adjusted quality of life by state (figure 3 & 4), we estimated a linear regression of the QoL score as outcome on a full set of state indicators (fixed effects) and 36 sex-specific age group (<45 years, 45-46, 46-47, 48-49, …, 69-70, 71-75, 76-80, 81-85, and 85+) indicators (fixed effects) with sample weights applied. Then, for each state, we averaged the predicted score of QoL if located in that state over all sample participants, i.e. the average adjusted prediction. This gave an estimate of what QoL score would be in a state if its age-sex composition was the same as that of the whole sample, which was representative of the national population aged 45+, when sample weights were applied.

We obtained analogous estimates for all outcomes i.e. pain prevalence (table 2) and QoL (table 4) and across sociodemographic characteristics. In this case, the logistic and linear regression from which the averaged predictions of the respective outcome were obtained included the categories of the sociodemographic characteristics and the 36 age-sex categories fixed effects.

References

1. Mohanty SK, Pedgaonkar SP, Upadhyay AK, Kampfen F, Shekhar P, Mishra RS, Maurer J, O'Donnell O: Awareness, treatment, and control of hypertension in adults aged 45 years and over and their spouses in India: A nationally representative cross-sectional study. PLoS Med 2021, 18(8):e1003740.
